# Ways to Build a Greener Healthcare System: Comment on "A Review of the Applicability of Current Green Practices in Healthcare Facilities"

**DOI:** 10.34172/ijhpm.9157

**Published:** 2025-06-11

**Authors:** Rui Wu, Yiming Liang

**Affiliations:** School of Business, Nanjing Normal University, Nanjing, China.

**Keywords:** Healthcare System, Carbon Footprint, Life Cycle Assessment, Hospital Sustainability, Circular Economy

## Abstract

Green practices have become the responsibility of healthcare system in the current warming planet. The article by Soares and colleagues reviews the literature on circular economy implementation in the European Union (EU) and its application in healthcare system. In this commentary, we complement the findings by discussing ways to build a greener healthcare system and introducing life cycle assessment (LCA), a method to quantify the environmental impacts of products and services in healthcare. LCA is useful to compare the environmental impacts of different clinical products and pathways. Within the healthcare system, avoiding overdiagnosis and overprescribing, improving building energy efficiency, and fleet electrification are important green practices. In addition, we cannot ignore the differences in regional energy system when comparing the carbon footprint of different healthcare systems.

## Introduction

 The review by Soares et al examines the practices of circular economy in the European Union (EU), including the policies formulated by the EU to reduce carbon emissions.^1^ Furthermore, this review focuses on the application of circular economy in healthcare facilities, especially green practices within hospitals across four dimensions: (1) health workforce, (2) water, sanitation and waste, (3) energy, and (4) infrastructure, technology and products. This commentary complements the findings by discussing several important ways to build a greener healthcare system. The year 2024 became the warmest on record globally, and it was announced as the first calendar year above 1.5 °C relative to pre-industrial levels by several international organizations.^2^ Climate change has increased the probability of extreme weather, affecting human survival and development. In this context, healthcare sector is also responsible for mitigating climate change, since reducing its environmental impact is in line with the sector’s goal of improving human health.

## Tackling Overdiagnosis and Overprescribing

 Globally, the increasing healthcare demand is the major driver of growth in healthcare-related carbon footprint and environmental impacts. The demand for healthcare is growing rapidly with the share of health expenditure of global gross domestic product from 8.6% in 2000 to 10.4% in 2021. Life expectancy is positively correlated with healthcare expenditures. However, countries with a life expectancy of 80 years at birth have a diminishing life expectancy benefit from their health expenditures. This means very high Human Development Index countries will spend more on healthcare than in the past to keep life expectancy increase. Although the health expenditure will continue to increase in the context of economic growth, there is room for improving effectiveness of healthcare expenditures. For instance, reducing the amount of unused medicines that have to be disposed of after reaching their expiration date is an effective approach to reduce the environmental impact of healthcare,^3^ since pharmaceuticals contribute significantly to the carbon footprint.^4,5^ Clinicians should also avoid overdiagnosis, such as ordering unnecessary imaging procedures (eg, magnetic resonance imagings [MRIs]). Following the principle of maximizing resource utilization efficiency, the circular economy prioritizes “reduce,” followed by “reuse” and “recycle.” In healthcare facilities, clinicians should prioritize avoiding overdiagnosis and overprescribing, in line with the first principle in circular economy of “reduce.”^6^ In addition, increasing the proportion of disease prevention can also improve effectiveness of healthcare expenditures by reducing the incidence of illness.

## Life Cycle Assessment of Medical Goods and Service

 “Reuse” is the second principle in circular economy. Unlike regular packaging, there are many additional treatments for the reuse of medical goods, such as cleaning, disinfection, and drying. Therefore, reusable medical products may not necessarily lead to better environmental performance than disposable products. Life cycle assessment (LCA) method is necessary to compare the environmental impacts of reusable and disposable products. LCA can assess the supply chain environmental impacts of medical products and services, which may not occur in healthcare facilities. The scope of the LCA will be determined by the goal of the assessment. To assess the environmental impacts of clinical use or procedure in healthcare facilities, the scope should be cradle-to-grave, including raw material extraction, material processing, part manufacturing, assembly, use, and end of life. The functional unit of the LCA should be defined for ensuring fair comparisons. In healthcare, the functional unit should be clinically oriented, focusing on patient-centered practices and outcomes, eg, one MRI scan. The functional unit includes all consumables and reusables. To assess one MRI scan, the data of environmental impacts for reusables, such as MRI scanner, should be scaled per use based on rated lifetimes of the scanner. Large amounts of data are required for LCA. The data may be collected by investigators or provided in an LCA inventory supplied by governments or commercial organizations. When the assessment scales up to an entire healthcare system, it is not realistic to compile a life cycle inventory of all goods within the system. For an assessment at the regional level, financial data along with emissions factors and input-output tables are needed. This approach is called Environmentally Extended Input Output analysis. Environmentally Extended Input Output links environmental impact with monetary quantity and calculates the life cycle impacts based on the matrix of monetary flows between economic sectors.^7-9^ In some regions, statistical departments do not provide input-output tables or emission factors containing separate healthcare or pharmaceutical sectors. Multiplying healthcare expenses by aggregated sectoral emission factor can lead to high uncertainty of the estimated life cycle emission. For example, pharmaceutical products, basic chemicals, fertilizers, and other chemicals are usually classified under the chemicals sector, but the carbon emission factor of this aggregated chemicals sector is much higher than that of the pharmaceuticals sector. Multiplying healthcare expenses by the emission factor of the aggregated chemicals sector may overestimate the carbon footprint of pharmaceuticals.

## Building and Travel Energy Use in Healthcare Facilities

 The staff in healthcare facilities have more control of the building and travel energy use than the energy use embodied in the procured goods. Like other public buildings, heating, ventilation, and air conditioning makes up a significant share of the total building energy consumption.^10^ To adapt to climate change, the demand for air cooling in hospitals has increased while the demand for heating has decreased. In the current and future decades, hospitals need to gradually adjust the timing of heating and cooling. This is not only about adapting to climate change, but also about saving energy and mitigating climate change. Utilizing hospital rooftop resources can improve building energy efficiency, such as installing photovoltaics and solar hot water heaters. In terms of travel energy use, electrification of hospital fleets can reduce greenhouse gas emissions, considering the gradual penetration of renewable energy generation. The energy management benefits from establishing a “green team” to develop interventions. The team should include clinicians, sustainability scientists or engineers, and administrative stakeholders.

## Participating in Regional Energy System Transformation

 The indirect energy use in healthcare facilities through the procurement of drugs and medical equipment is much larger than the building and travel energy use. However, the indirect energy use is beyond the control of healthcare system. Therefore, the carbon footprint and environmental impacts of healthcare facilities are largely determined by the regional energy system. The regions with a cleaner energy system are more likely to have a lower carbon intensity of healthcare expenditure.^11^ The technological progress and policies in energy sectors and energy-intensive sectors determine the energy system transformation. Although the healthcare sector, not an energy intensive one, has limited impact on the low-carbon transformation of energy systems, the staff in this sector can still participate in the transformation. For example, hospitals can purchase Green Power Certificate to support carbon neutrality. A Green Power Certificate, also known as a Renewable Energy Certificate in some regions, is a market-based instrument for businesses and individuals to reduce their carbon footprint without needing direct access to green power.

## Conclusion

 Green practices in healthcare facilities are receiving increasing attention worldwide. Circular economy is a concept allows the sustainable development of healthcare and LCA is an important approach to achieve the target. The review conducted by Soares et al contribute importantly to the process of consolidating and integrating the different aspects of green practices in healthcare facilities, identifying where there is consensus and where further work is needed.

## Ethical issues

 Not applicable.

## Conflicts of interest

 Authors declare that they have no conflicts of interest.
